# Identification of TREM2-positive tumor-associated macrophages in esophageal squamous cell carcinoma: implication for poor prognosis and immunotherapy modulation

**DOI:** 10.3389/fimmu.2023.1162032

**Published:** 2023-04-28

**Authors:** Hongmu Li, Yu Miao, Leqi Zhong, Songjie Feng, Yue Xu, Lu Tang, Chun Wu, Xianzhou Zhang, Ling Gu, Hengyi Diao, Huiyun Wang, Zhesheng Wen, Minglei Yang

**Affiliations:** ^1^ Department of Thoracic Surgery, Sun Yat-sen University Cancer Center, Guangzhou, China; ^2^ State Key Laboratory of Oncology in South China, Sun Yat-sen University Cancer Center, Guanghzou, China; ^3^ College of Life Sciences, University of Chinese Academy of Sciences, Beijing, China; ^4^ Max F. Perutz Laboratories, Medical University of Vienna, Vienna, Austria; ^5^ Department of Pathology, First Affiliated Hospital of Zhengzhou University, Zhengzhou, China; ^6^ Department of Hepatobiliory and Pancreatic Surgery, Henan Provincial Cancer Hospital, Zhengzhou, China; ^7^ Zhongshan School of Medicine, Sun Yat-sen University, Guangzhou, China

**Keywords:** tumor-associated macrophages, esophageal squamous cell carcinoma, prognosis, single-cell RNA sequencing, immunotherapy modulation

## Abstract

**Background:**

It is now understood that the effectiveness of checkpoint immunotherapy can be impaired by immunosuppressive tumor-associated macrophages (TAMs). Nonetheless, the impact of different TAM subpopulations on the antitumor immune response remains unclear, mainly due to their heterogeneity. Herein, we identified a novel TAM subpopulation in esophageal squamous cell carcinoma (ESCC) that might contribute to poor clinical outcomes and immunotherapy modulation.

**Methods and results:**

We analyzed two single-cell RNA sequencing (scRNA-seq) datasets (GSE145370 and GSE160269) of esophageal squamous cell carcinoma to identify a novel TREM2-positive TAM subpopulation characterized by upregulation of *TREM2, C1QC, C1QB, C1QA, SPP1, and APOE*. Quantitative real-time PCR (qRT-PCR) and enzyme-linked immunosorbent assay (ELISA) demonstrated that these genes were significantly overexpressed in ESCC. Multiplex immunofluorescence validated the infiltration of TREM2^+^ TAMs in ESCC tissues, which correlated with poorer overall survival (OS). The scRNA-seq analysis in dataset GSE120575 indicated significant enrichment of TREM2^+^ TAMs in melanoma patients (n=48) with poor immunotherapy response, which had an identical gene signature with TREM2^+^ TAMs from ESCC. Analysis of 29 bulk-RNA melanoma samples from dataset GSE78220 revealed that a gene signature of 40 genes associated with TREM2^+^ TAMs was upregulated in the transcriptome of melanomas that did not respond to anti-PD1 therapy. Validation in the TCGA ESCC cohort (n=80) showed that a high enrichment score of the TREM2^+^ TAM was associated with poor prognosis. In addition, 10 ESCC patients treated with anti-PD1 therapy suggested that patients who are not sensitive to immunotherapy have higher density of TREM2+TAMs infiltration.

**Conclusion:**

Overall, TREM2^+^ TAM infiltration in ESCC is associated with poor prognosis and may serve as a biomarker for predicting outcomes and immunotherapy modulation in this patient population. modulation; single-cell RNA sequencing

## 1 Introduction

Esophageal cancer (ESCA) is the eighth most common cancer type and the sixth leading cause of cancer death worldwide. It has been established that esophageal squamous cell carcinoma (ESCC) accounts for approximately 90% of all ESCA (1). Despite significant inroads achieved in multidisciplinary treatment, the prognosis of patients with ESCC remains dismal (2). In recent years, immune checkpoint inhibitors have gained considerable momentum, especially in the field of immunotherapy (3). There is an increasing consensus that monoclonal antibody (mAb) inhibition of programmed death 1 (PD1) or programmed death ligand 1 (PD-L1) can yield convincing responses and clinical advantages across numerous malignancies, including ESCC (4, 5). However, only a subset of patients responds to treatment (6). Therefore, exploring the causes of immunotherapy resistance and finding new immunotherapy targets is necessary.

Tumor-associated macrophages (TAMs) are widely acknowledged to be involved in tumorigenesis, progression, angiogenesis, and metastasis (7, 8). In addition, high TAMs infiltration has been associated with poor clinical outcomes in various tumors and has long been thought to reduce response to therapy, including radiation therapy, chemotherapy, and immunotherapy (9–14). However, due to the intricate heterogeneity of TAMs, their application in tumor treatment is limited (7, 15). Single-cell RNA sequencing (scRNA-seq) techniques may help differentiate more TAM subpopulations and facilitate the development of more effective therapeutic strategies (16).

Yonatan et al. (17) used intracellular staining and sequencing integrated technology and defined a suppressor myeloid cell population specifically overexpressing TREM2, which could promote T cell dysfunction and tumor immune escape. When the *TREM2* gene was ablated, the abundance of regulatory myeloid immune cells and exhausted CD8*^+^* T cells was significantly reduced, and tumor growth was inhibited. Similarly, Martina et al. (18) reported that *Trem2* ^-/-^ mice were more resistant to the growth of various cancers and more sensitive to anti-*PD1* immunotherapy. Furthermore, anti-*TREM2* mAb combined with anti-*PD1* inhibited tumor growth and promoted regression, demonstrating that anti-*TREM2* therapy may broaden the arsenal of myeloid cell targeting in tumors. Nevertheless, the role of the TREM2^+^ myeloid cell population in ESCC has been largely underexplored.

Herein, we identified and validated a novel TREM2^+^ TAM subpopulation from two scRNA-seq ESCC datasets and revealed its molecular characteristics. Interestingly, scRNA-seq analysis of melanoma patients treated with immunotherapy demonstrated that TREM2^+^ TAMs were linked to immunotherapy modulation, which exhibited a similar gene signature to TREM2^+^ TAMs in ESCC. After analyzing the gene expression patterns of 80 cases of ESCC from The Cancer Genome Atlas (TCGA), we validated the correlation of this subgroup with unfavorable prognosis and resistance to immunotherapy.

## 2 Materials and methods

### 2.1 Dataset collection

Two single-cell transcriptome datasets of patients with ESCC were obtained from the NCBI Gene Expression Omnibus (GEO) database (https://www.ncbi.nlm.nih.gov/, GSE145370 (19) and GSE160269 (20)). The GSE160269 dataset contained tumor (n=60) and adjacent normal (n=4) tissue samples, and the GSE145370 dataset comprised tumor (n=7) and adjacent normal (n=7) tissue samples. To identify novel cell types, dataset GSE160269 was used as the training dataset and GSE145370 as the validation dataset. Multi-omics datasets of 80 patients with ESCC, including bulk RNA-seq, somatic mutation, copy number alteration, and clinicopathological parameters, were retrieved from TCGA Data Portal (https://tcga-data.nci.nih.gov/tcga/). To validate the association of immunotherapy response, the scRNA-seq data and clinical information of 48 tumor samples biopsied from 32 metastatic melanoma patients treated with checkpoint therapy were downloaded from GEO under accession GSE120575 (21). In addition, GEO accession GSE78220 was used to acquire clinical information and bulk RNA-seq data from 29 melanoma patients who received anti-PD1 treatment (22).

### 2.2 Single-cell RNA-seq data analysis for ESCC

We used the same pipeline to conduct scRNA-seq analysis of GSE160269 and GSE145370. Quality control was performed on the raw count matrix of each scRNA-seq sample using Seurat v4.1 (23). The cells containing >20% mitochondrial genes and <200 and >6000 expressed genes were considered low quality and were excluded from further analysis. The top 2000 highly variable genes for dataset integration were identified by the FindVariableFeatures function. The datasets from all samples were integrated with the CCA method wrapped in Seurat to remove batch effects. We scaled the data using the ScaleData function and performed principal component (PC) analysis using the RunPCA function. The top 50 PCs were used for single-cell clustering analysis with the FindNeighbors and FindClusters functions. The critical parameter “resolution” was set to 1 for a more refined result. Preliminary annotation was performed in each cell cluster using SingleR (24), an automatic annotation tool. To obtain more accurate cell annotation results, we manually checked the expression levels of known classical marker genes in each cell type annotated by SingleR and corrected some cell annotations. Based on *TREM2*, *APOE*, *SPP1*, *C1QC*, *C1QA*, and *C1QB* expression levels, we identified a novel TAM subpopulation and defined it as TREM2*^+^* TAMs. The cell clusters were embedded into two-dimensional space and visualized by Uniform Manifold Approximation and Projection (UMAP) with the top 50 PCs. Differential gene expression was analyzed using the FindAllMarkers function with default parameters to identify the marker genes for each cell type. Differentially expressed genes (DEGs) of the novel TAM subpopulation between tumor and adjacent normal tissue were identified using the FindMarkers function. Processes and pathway enrichment on the DEGs were analyzed using Metascape (http://metascape.org), a web-based application (25).

We calculated the cell proportion for each cell type in each tumor and adjacent normal tissue sample and conducted a Wilcoxon rank-sum test on the proportion of each cell type between the tumor and adjacent normal tissues to identify significant differences in cell proportions.

We compared the top 50 highly expressed genes of TREM2^+^ TAMs between datasets GSE145370 and GSE160269 and identified 40 overlapping genes defined as the TREM2^+^ TAM gene signature. Then, ClueGO/CluePedia (26) was applied to interpret the immunological function of the TREM2^+^ TAM gene signature with the immune system process gene ontology terms.

### 2.3 Cell-cell communication analysis

Given that dataset GSE160269 contained more tumor samples than GSE145370, we selected it for further analysis. To explore the underlying intercellular networks in the ESCC TME, we extracted all cells derived from tumor samples for cell-cell communication analysis using CellChat (27). Secreted Signaling, a subset of CellChatDB, was used to calculate the aggregated cell-cell communication network and identify overexpressed ligand-receptor interactions mediating intercellular communication.

### 2.4 Correlating TREM2^+^ TAMs with immunotherapy response in melanoma

To investigate the association of TREM2*^+^* TAMs with immunotherapy response, we re-analyzed the scRNA-seq data of 48 melanoma samples treated with ICB therapy. The quality control, cell clustering, and cell type annotation were performed with the same criteria described above. We utilized the top 23 principal components to create UMAP plots for visualizing cell clusters in two-dimensional space. From these plots, we identified TREM2+ tumor-associated macrophages significantly enriched in melanomas that did not respond to immune checkpoint blockade therapy. To further investigate whether the expression pattern of TREM2*^+^* TAMs from ESCC was consistent with that of TREM2*^+^* TAMs from melanoma, we extracted macrophages of ESCC and melanoma, integrated these macrophages using Harmony and clustered them using UMAP with 18 Harmony embeddings. Finally, we utilized publicly available bulk RNA-seq data from 29 melanoma patients who received anti-PD1 treatment to establish the relationship between the top 50 gene signatures of TREM2+ tumor-associated macrophages in ESCC and resistance to immune checkpoint blockade therapy (22).

### 2.5 Identification of a high TREM2^+^ TAM-enriched subset of patients with ESCC

To investigate how TREM2+ tumor-associated macrophages exert a suppressive effect on the immune response against tumors, we employed the gene signature of these TAMs that was identified during single-cell analysis as representative of this cell type. Using this gene signature, we calculated the enrichment score of TREM2*^+^* TAMs for 80 patients with ESCC *via* single-sample gene set enrichment analysis (ssGSEA) (28) in gene set variation analysis (GSVA) (29) in R. The immune and stromal enrichment scores were calculated by ssGSEA with two gene signatures from the study conducted by Yoshihara (30). We also collected gene sets characterizing Tex, immunosuppressive cell signatures, and Tex-related signaling pathways: (1) Tex gene sets: CD8 Tex profiled by mass cytometry (CyTOF) (*CD8*_Tex_CyTOF) (31), human gene sets homologous to CD4 Tex (*CD4*_Tex_Mouse) and CD8 Tex (*CD8*_Tex_Mouse) in mice with chronic viral infection (32), CD8 TEX in hepatocellular carcinoma (*CD8*_Tex_HCC) (33), and CD8 Tex in patients with melanoma (*CD8*_Tex_Melanoma) (21); (2) immunosuppressive cells: macrophages, myeloid-derived suppressor cell (34) and CD4 regulatory T cells (Tregs) (35); and (3) MSigDB signaling pathways (https://www.gsea-msigdb.org/gsea/msigdb): TGF_BETA_SIGNALING, TNFA_SIGNALING_VIA_NFKB, INTERFERON_GAMMA_RESPONSE, and INTERFERON_ALPHA_RESPONSE. For each gene set or signature, the enrichment scores of the patients with ESCC were calculated by ssGSEA. The resultant enrichment score was converted into z-scores among the ESCC cohort and then transformed to 0-1 *via* the sigmod function. Then, we performed hierarchical clustering analysis on the 80 patients with ESCC using the sigmod-transformed enrichment scores. Finally, the patients were ranked according to the TREM2*^+^* TAMs enrichment scores to identify the high TREM2*^+^* TAMs-enriched group, hereafter referred to as the “High abundance group”.

### 2.6 Cellular and molecular characterization of the High abundance group

The absolute proportions of immune cells between the high and low TREM2^+^ TAMs-enriched groups (High and Low abundance groups, respectively) were quantified and compared by the CIBERSORT algorithm (36). To identify the DEGs between the two groups, the read count data of the 80 patients with ESCC were downloaded from TCGA. Differential expression analysis was performed using DESeq2, and genes with false discovery rates (FDR) < 0.05 and log2 fold change (log2FC) > 1 were considered statistically significant (37). Unbiased GSEA was performed using Kyoto Encyclopedia of Genes and Genomes (KEGG) pathways and MSigDB hallmark gene sets to identify activated pathways and hallmark gene sets enriched in the High abundance group *via* clusterProfiler (38).

### 2.7 Prediction of immune checkpoint blockade therapy response

The Tumor Immune Dysfunction and Exclusion (TIDE) algorithm (39) was used to predict potential ICB therapy response based on the gene expression profiles of the 80 patients with ESCC. We compared the TIDE scores of the High and Low abundance groups.

### 2.8 Quantitative real-time PCR

The expression profiles were examined by quantitative qRT-PCR to obtain accurate results. This study was approved by the Sun Yat-sen University Cancer Center Ethics Committee (GZR2018-120). The samples were derived from 22 patients who underwent ESCC radical surgery at our center. The total RNA was isolated from ESCC and adjacent normal tissue samples using the TRIzol reagent (TIANGEN, Beijing, China) and reverse-transcribed to complementary DNA using PrimeScript™ RT Master Mix (ES Science, Shanghai, China). The qRT-PCR was conducted using SYBR Green Master Mix (ES Science, Shanghai, China). The qRT-PCR assays were conducted in triplicate in 10-mL reaction volumes for each sample. *GAPDH* served as the internal control to normalize hub gene expression. The relative expression was calculated using the comparative threshold cycle (2-Ct) method.

### 2.9 Enzyme-linked immunosorbent assay

Blood samples were obtained from 14 untreated patients with ESCC and 10 healthy donors after obtaining the approval of the Sun Yat-sen University Cancer Center ethics committee (GZR2018-120). All blood samples were collected and rapidly centrifuged to collect the plasma, and samples were stored in an ultralow-temperature refrigerator at 80°C. All samples were collected following the ethical guidelines of Sun Yat-sen University Cancer Center, and all participants provided informed consent. The expression of *TREM2*, *SPP1*, *APOE*, *C1QC*, *C1QB*, and *C1QA* (i.e., the complement *CIQ* genes) was quantified using a 96T human ELISA kit (Jiangsu Meimian Industrial Co., Ltd., Jiangsu, China).

### 2.10 Multiplex immunofluorescence staining

Multiplex immunofluorescence staining was employed to identify the new TAM subpopulation using formalin-fixed paraffin-embedded (FFPE) tissues from 45 ESCC patients. Two consecutive rounds of staining were carried out on each FFPE tumor section. The primary antibodies used in this study include *CD68* Rabbit polyclonal antibody (1:2400, Cell Signaling Technology, Shanghai, China) and *TREM2* Rabbit polyclonal antibody (1:1000, Proteintech, Suzhou, China). The stained signal was performed according to the manufacturer’s protocol using the PDOne TSA-RM-8275 kit (PANOVUE, Guangzhou, China) and EDTA (PH=9) repair liquid was also used. After two rounds of staining, the sections were counterstained with DAPI (PANOVUE, Guangzhou, China). Multiple stained sections were imaged by Polaris Automatic Digital Slide Scanner, and the abundance of novel TAM was analyzed by Phenochart software. Finally, the proportion of stained parts was used as the scoring basis.

### 2.11 Statistical analysis

Data processing and plotting in this study were performed in GraphPad Prism 8.0.1 (San Diego, California, USA) and R 3.5.1 (http://www.r-project.org). Using the Wilcoxon rank-sum test for continuous data, we examined the correlation between the High and Low abundance groups based on copy number alteration and mutation number. Overall survival (OS) and progression-free survival (PFS) were analyzed using Kaplan–Meier estimates and log-rank testing. Two- or one-tailed *P* < 0.05 was considered statistically significant.

## 3 Results

### 3.1 Identification of TREM2^+^ TAMs by single-cell RNA-seq analysis

**Figure 1:**
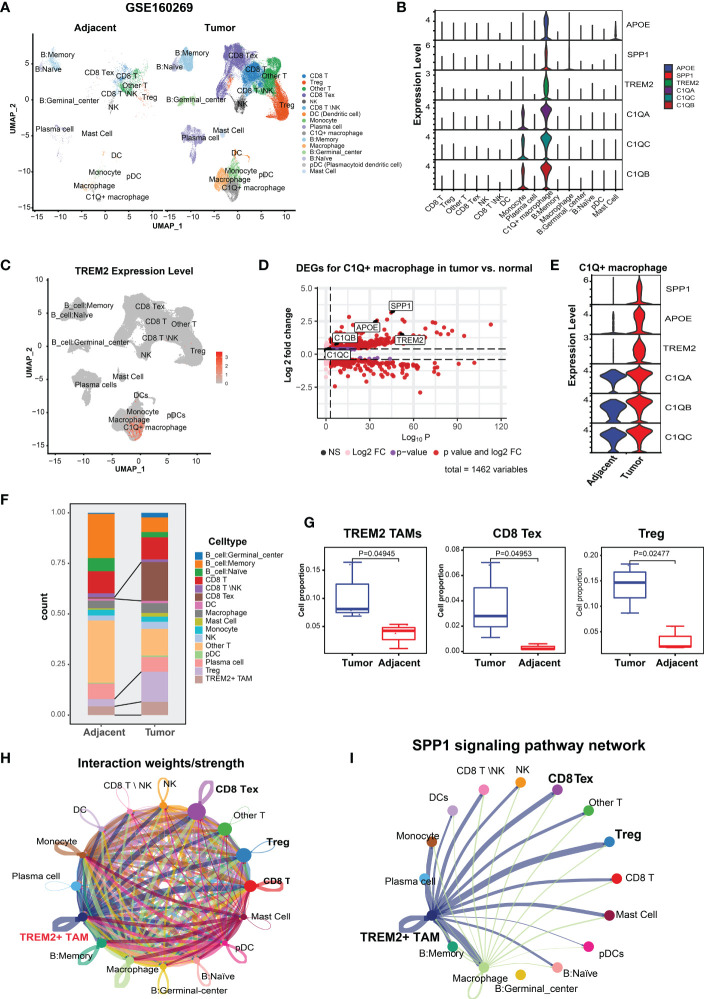
Identification and characterization of TREM2^+^ TAMs in ESCC by scRNA-seq analysis (GSE160269). **(A)** UMAP plot of cells colored by cell types and splitted by tumor and adjacent normal tissue. **(B)** Violin plot showing expression level of *APOE*, *SPP1*, *TREM2*, *C1QA*, *C1QC*, and *C1QB* among 16 cell types. **(C)** UMAP plot showing expression level of TREM2 in each cell type. **(D)** Volcano plot showing differentially expressed genes of C1Q^+^ macrophage in tumor vs. adjacent normal tissue. **(E)** Violin plot showing expression level of *SPP1*, *APOE*, *TREM2*, and three complement genes in C1Q^+^ macrophage between tumor and adjacent normal tissue. **(F)** Cell proportion of each cell type in tumor and adjacent norm tissue. **(G)** Cell proportion comparison of TREM2^+^ TAMs, CD8 Tex and Treg between two group, each dot represents a sample. **(H)** Cell-cell interaction network generated by CellChat. **(I)** Cell-cell communication network *via* SPP1 signaling pathway.

We analyzed the scRNA-seq data of CD45^+^ cells deposited in GSE160269, which included 60 ESCC tumor and 4 adjacent normal tissue samples. Cells that passed quality control (110,980) were clustered and assigned to 16 cell types, which included CD8 T cells, Tregs, CD8 Tex, NK cells, dendritic cells, and macrophages (**Figure 1A**). The top 100 highly expressed genes (average log2FC > 0.25, *P* < 0.05) of these cell types were summarized in **Supplementary Table S1**. We focused on TAMs and discovered a novel macrophage subpopulation with higher expression of *C1QA*, *C1QB*, *C1QC*, *TREM2*, *SPP1*, and *APOE* (**Figure 1B**). Differential expression analysis for all cell types revealed that *C1QA*, *C1QB*, *C1QC*, *SPP1*, and *APOE* were the top five highly expressed genes (**Supplementary Table S1**). Furthermore, >84% of the cells in this novel macrophage subpopulation expressed the three complement *C1Q* genes (i.e., *C1QA*, *C1QB*, *C1QC*) (**Supplementary Figure S1A**, **Supplementary Table S1**), while 52.3%, 66.3%, and 70.6% of the cells expressed *SPP1, APOE*, and *TREM2* (**Figure 1C**, **Supplementary Figure S1A**, **Supplementary Table S1**), respectively. Subsequently, this subpopulation was referred to as *C1Q^+^* macrophages. As expected, <5% of the cells in the remaining populations expressed these genes (**Supplementary Table S1**). Comparison of the DEGs of *C1Q^+^* macrophages between the tumor and adjacent normal tissue revealed that tumor *C1Q* ^+^ macrophages exhibited higher expression of these six genes (average log2FC > 0.25, *P* < 0.05) (**Figure 1D**). Nonetheless, *SPP1, APOE, and TREM2* manifested more average fold changes than the C1Q genes and were expressed in more tumor cells than normal tissue cells (**Figure 1E**; **Supplementary Table S2**). Metascape (25) pathway and process enrichment analysis of the DEGs in the *C1Q^+^* macrophages between tumor and normal tissue demonstrated that the cytokine-related pathways were enriched in tumor *C1Q* ^+^ macrophages (**Supplementary Figure S1B**). These results substantiated that *C1Q* ^+^ macrophages with high expression levels of *SPP1*, *APOE*, and *TREM2* were associated with an immunosuppressive TME. Multiple studies have identified similar TAM subpopulations: single-cell protein activity analysis in renal tumors revealed that the TAM population with upregulation of *APOE*, *TREM2*, and the three *C1Q* genes was associated with recurrence (40). In a mouse tumor model, TAM-expressed *TREM2* was associated with increased CD8 Tex and immunotherapy resistance and targeting *TREM2* on TAMs synergized with anti-*PD1* immunotherapy (18, 41, 42). Therefore, we defined the tumor *C1Q^+^* macrophages as TREM2*^+^* TAMs.

Next, we investigated the TME cellular components to understand the impact of TREM2^+^ TAMs on immunosuppression. CD8 Tex with elevated expression of multiple inhibitory receptors (*PDCD1*, *CTLA4*, *LAG3*, *TIGIT*, *HAVCR2*) and Tregs with high expression levels of *FOXP3*, *TIGIT*, and *CTLA4* (**Supplementary Figure S1C**) were enriched in the ESCC TME (**Figure 1F**), which contributed to tumor immune escape and immunotherapy resistance (39, 43). Comparison of the abundance of each cell type between 8 paired tumor and normal samples revealed that the tumor tissue had significantly higher proportions of CD8 Tex, Tregs, and TREM2^+^ TAMs (**Figure 1G**), suggesting that TREM2*^+^* TAMs were related to increased CD8 Tex and contributed to an immunosuppressive TME, consistent with observations in mouse tumor models (18).

An increasing body of evidence suggests that mediated by ligand-receptor interactions, intercellular communication is vital in suppressing the antitumor immune response and T cell exhaustion (44, 45). Based on the analysis of intercellular communication between immune cells in the ESCC TME using CellChat (34), TREM2^+^ TAMs were predicted to have a higher overall interaction strength with CD8 T cells, CD8 Tex, and Tregs (**Figure 1H**). We also determined that TREM2*^+^* TREM2+ TAMs exhibited a greater likelihood of communication with CD8 Tex or Tregs, compared to other cell types, through SPP1-related signaling pathways (**Figure 1I**), specifically SPP1–CD44 and SPP1–(ITGA4+ITGB1) (**Supplementary Figure 1D**). Overall, we identified a novel TREM2^+^ TAMs in ESCC that was associated with Tex and contributed to an immunosuppressive TME.

### 3.2 Validation of the presence of TREM2^+^ TAMs by independent scRNA-seq data

**Figure 2:**
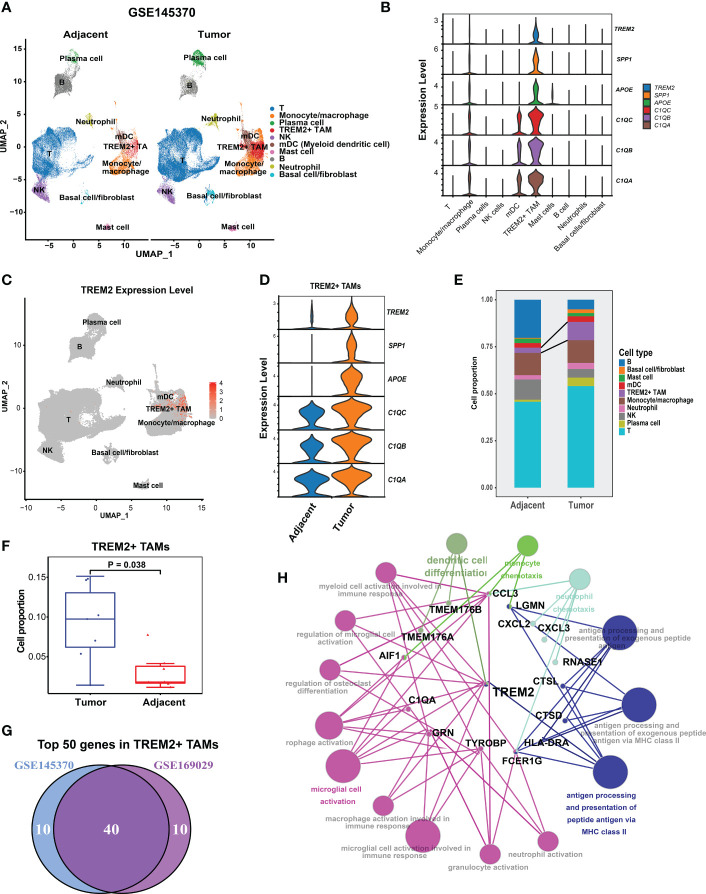
Validation of presence of TREM2^+^ TAMs by GSE145370.**(A)** UMAP plot of cells colored by cell types and splitted by tumor and adjacent normal tissue. **(B)** Violin plot showing expression level of *APOE, SPP1, TREM2, C1QA, C1QC*, and *C1QB* among 10 cell types. **(C)** UMAP plot showing expression level of TREM2 in each cell type. **(D)** Violin plot showing expression level of of *SPP1*, *APOE*, *TREM2*, and three complement genes in TREM2^+^ TAMs between tumor and adjacent normal tissue. **(E)** Cell proportion of 10 cell types in tumor and adjacent normal tissue. **(F)** Cell proportion comparison of TREM2^+^ TAMs between two group, each dot represents a sample using Wilcoxon rank-sum test. **(G)** Venn plot showing 40 overlapping genes of top 50 highly expressed genes in TREM2^+^ macrophage between GSE145370 and GSE169029. **(H)** The functionally grouped network of immune system process gene ontology terms by ClueGO/CluePedia for the interpretation of TREM2+ TAM gene signature’s biological roles. Small red dots represent genes and colour nodes represent immune system process gene ontology terms. Node colours represent distinct functional groups. Node size represents the significance of the terms. The more significant the terms are, the larger highlighted nodes.

To validate the presence of TREM2*^+^* TAMs, we analyzed the ESCC scRNA-seq data of GSE145370 (19) using the same pipeline. Quality control yielded 112,714 cells and clustered them into 10 cell types (**Figure 2A**). We focused on macrophages and identified the TREM2*^+^* TAMs that also highly expressed *TREM2*, *SPP1*, *APOE*, *C1QA*, *C1QB*, and *C1QC* (**Figure 2B**). Notably, differential expression analysis for each cell type determined that the top six highly expressed genes of TREM2*^+^* TAMs contained *SPP1*, *APOE*, *C1QA*, *C1QB*, and *C1QC* (**Supplementary Table S3**), consistent with the TREM2*^+^* TAMs in GSE160269. Similarly, >75% of cells in the TREM2*^+^* TAM population expressed *C1QA*, *C1QB*, and *C1QC*, and 64.6%, 49.8%, and 54.4% of the cells expressed *APOE*, *SPP1*, and *TREM2*, respectively (**Figure 2C**, **Supplementary Table S3**). Comparison of the gene expression of TREM2*^+^* TAMs between the tumor and adjacent normal tissue revealed that *TREM2*, *SPP1*, APOE, *C1QA*, *C1QB*, and *C1QC* were upregulated in tumors (**Figure 2D**, **Supplementary Table S4**), which was consistent with the results for GSE160269. Regarding changes in cell composition, the tumor samples had significantly higher cell proportions of TREM2*^+^* TAMs than the adjacent normal tissue (**Figures 2E, F**). A primary investigation of this data indicated that CD8 Tex and Tregs were abundant in the tumor (19). In addition, a comparison of the top 50 highly expressed genes of TREM2*^+^* TAMs between GSE145370 and GSE160269 revealed 40 overlapping genes (**Figure 2G**). Then we defined the 40 overlapping genes as the TREM2^+^ TAMs gene signature. The functionally grouped network of immune system process gene ontology analyzed by ClueGO showed that the TREM2^+^ TAMs gene signature was significantly associated with macrophage activation and antigen processing-related immunological functions (**Figure 2H**).

Taken together, we successfully identified and validated a novel TAM subpopulation (TREM2*^+^* TAM) associated with Tex and contributes to an immunosuppressive TME in ESCC. *TREM2*, *SPP1*, *APOE*, *C1QA*, *C1QB*, and *C1QC* were the signature genes of the TREM2*^+^* TAMs, among which the TREM2 receptor on TAM was a therapeutic target when combined with ICB to enhance the antitumor response (18).

### 3.3 Validation of specific signature genes of TREM2^+^ TAMs

**Figure 3:**
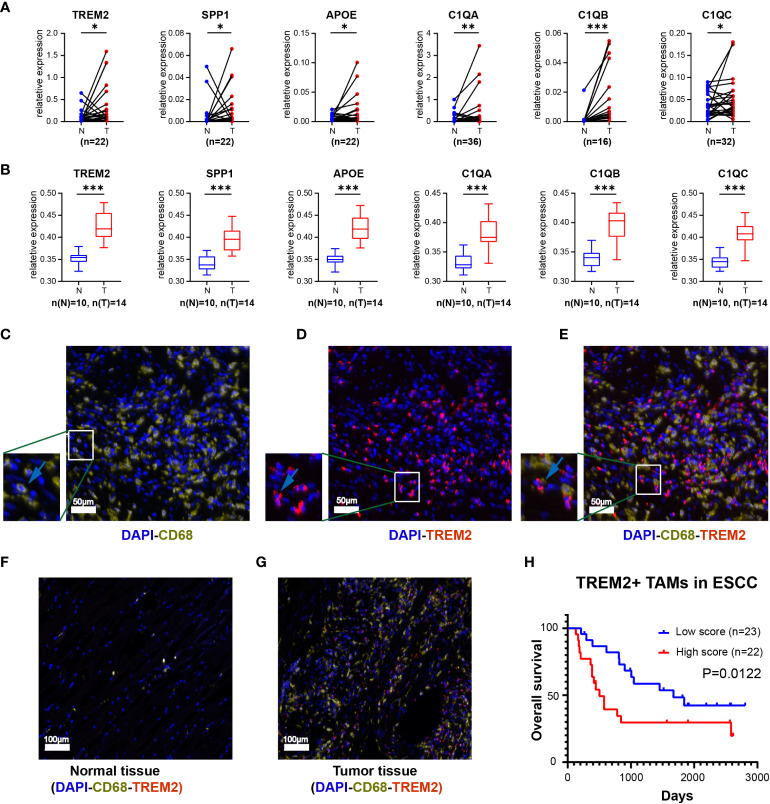
The expression level of top 6 genes of Novel TAM subpopulation between ESCC tissue and normal tissue. **(A)** Expression levels of *TREM2*, *SPP1*, *APOE*, *C1QA*, *C1QB*, and *C1QC* in ESCC tissues and corresponding normal tissues using qRT-PCR. **(B)** Protein expression levels of TREM2, SPP1, APOE, C1QA, C1QB, and C1QC in the plasma of ESCC patients and healthy controls. **(C-E)** Three-color multispectral and separated individual spectral images of multiplexed immunofluorescence staining with DAPI (blue), CD68 (yellow), and TREM2 (red). **(F)** TREM2^+^ TAMs in normal esophageal tissue. **(G)** TREM2^+^ TAMs in tumor esophageal tissue. **(H)** ESCC having high levels of TREM2^+^ TAMs had the worst OS (P = 0.0122). *, P <0.05; **, P < 0.01; ***, P < 0.001.

Kaplan-Meier Plotter (https://kmplot.com/analysis/) and Gene Expression Profiling Interactive Analysis 2 (GEPIA2, https://gepia2.cancer-pku.cn/) online database were used to verify the expression of the specific signature genes of the TREM2*^+^* TAM subpopulation, which demonstrated *TREM2*, *SPP1*, *APOE*, *C1QC*, *C1QB*, and *C1QA* were highly expressed in esophageal cancer (**Supplementary Figure S2**) and correlated with poor prognosis (**Supplementary Figure S3**). Similarly, qRT-PCR yielded consistent results for the ESCC samples from our center (**Figure 3A**, **Supplementary Table S5**). The expression of the proteins encoded by the signature genes of the TREM2^+^ TAMs was analyzed by the Human Protein Atlas database (https://www.proteinatlas.org) (46). We found that the signature genes of the TREM2^+^ TAMs encode secretory proteins. Then ELISA was used to examine the plasma expression levels of each protein between the patients with ESCC and healthy donors. The levels of TREM2, SPP1, APOE, C1QC, C1QB, and C1QA were higher in the plasma of patients as compared to that of healthy donors (**Figure 3B**, **Supplementary Table S6**).

To assess the infiltration of TREM2*^+^* TAMs in tumor tissues, we labeled macrophages with CD68 and subsequently observed the expression of TREM2 on this cell type (**Figures 3C-E**). Multiplex immunofluorescence showed that TREM2*^+^* TAMs were scarce in normal tissues but abundant in tumor tissues (**Figures 3F, G**). The survival analysis revealed that the overall survival (OS) of the high TREM2^+^ TAMs abundance group was poor (*p = 0.0122*) based on FFPE tissue from 45 ESCC patients (**Supplementary Table S7**, **Figure 3H**).

### 3.4 The same TREM2^+^ TAMs enrichment associated with immunotherapy resistance was found in melanomas

**Figure 4:**
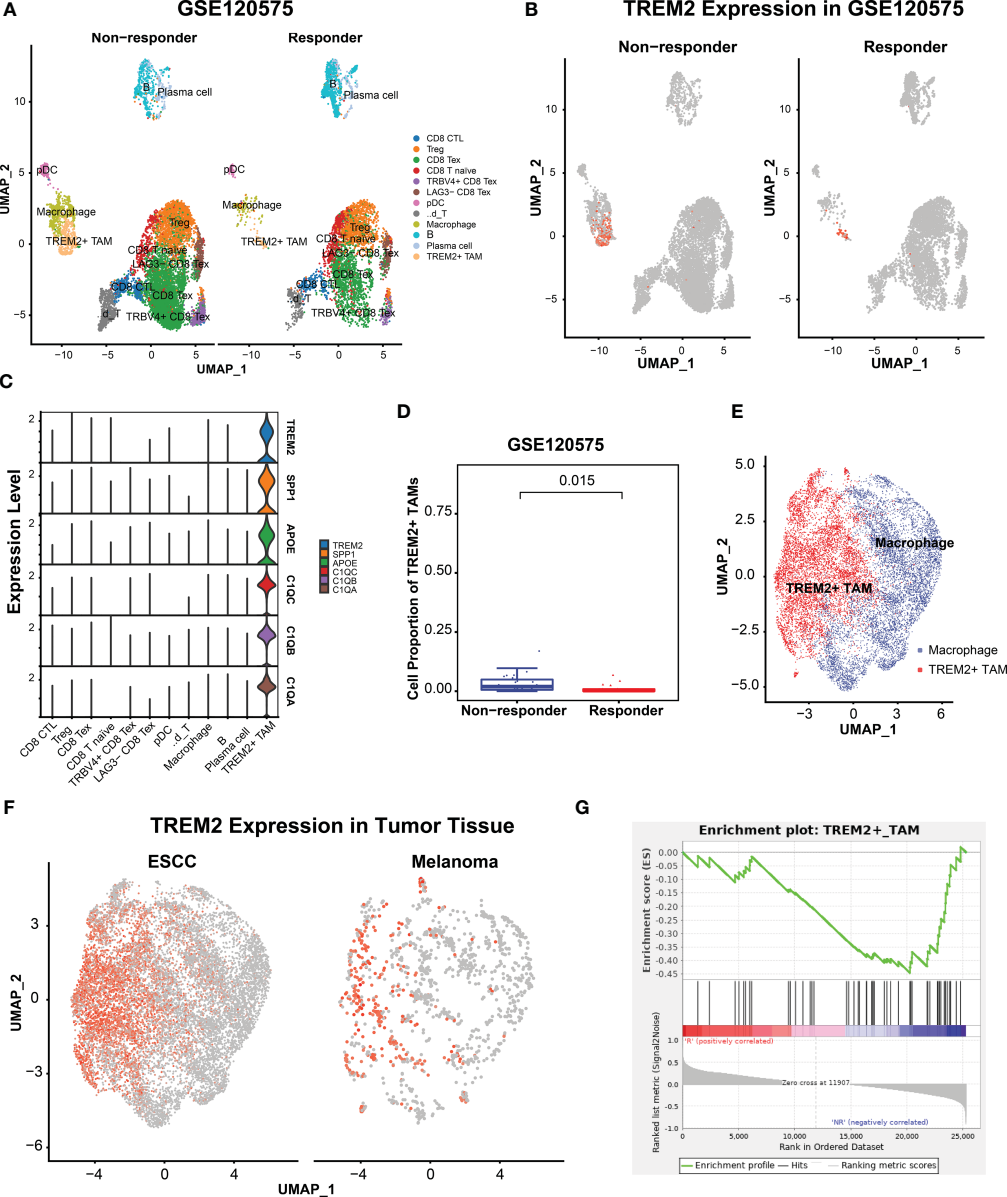
TRME2^+^ TAMs enriched in melanomas with no response to immunotherapy. **(A)** UMAP plot of cells colored by cell types and splitted by responder and non-responder using GSE120575. **(B)** UMAP plot of cells colored by expression level of *TREM2*. **(C)** Violin plot showing expression level of *APOE*, *SPP1*, *TREM2*, *C1QA*, *C1QC*, and *C1QB* among 12 cell types. **(D)** Boxplot showing cell proportion of TREM2^+^ TAMs between responder and non-responder. Wilcoxon test was used. **(E)** UMAP plot of macrophages from ESCC and melanoma. Red dots indicate TREM2^+^ TAMs, Other dots indicate the rest macrophages. **(F)** UMAP plot of macrophages colored by TREM2 expression level and splitted by ESCC and melanoma. **(G)** Gene set enrich analysis on the RNA-seq profiles of 25 melanoma patients treated with anti-PD1 immunotherapy. R represented response to anti-PD1, NR represented no response.

To investigate the association of TREM2*^+^* TAMs with immunotherapy resistance, we re-analyzed scRNA-seq data of 48 melanomas (GSE120575) treated with ICB therapy. After quality control, 16127 cells were retained and clustered into 12 cell types, including *CD8* cytotoxic T lymphocytes (*CD8* CTL), Treg, *CD8* exhausted T cells (*CD8* Tex), *CD8* T naïve cells, *TRBV4^+^ CD8* Tex, *LAG3- CD8* Tex, plasmacytoid dendritic cells, γδ T cell, macrophages, B cells, plasma cells and TREM2*^+^* TAMs (**Figure 4A**). The top 100 highly expressed genes of each cell type are listed in **Supplementary Table S8**. We focused on macrophages and identified TREM2*^+^* TAMs (**Figure 4B**) specifically expressing *TREM2, SPP1, APOE, C1QC, C1QB*, and *C1QA* (**Figure 4C**), which were significantly upregulated in melanomas not responsive to ICB therapy (**Figure 4D**). As expected, TREM2*^+^* TAMs from ESCC specifically expressed *TREM2, SPP1, APOE*, and three complement genes, consistent with TREM2*^+^* TAMs from melanomas. To validate the differences in gene expression patterns of TREM2*^+^* TAMs between ESCC and melanomas, we extracted the macrophages from the two tumors and integrated them into a shared dimensional space. Then we performed clustering and visualization with UMAP plots (**Figure 4E**). We also observed that TREM2*^+^* TAMs from ESCC and melanomas were clustered together (**Figure 4F**), suggesting a consistent expression pattern of TREM2*^+^* TAMs between ESCC and melanomas. Moreover, we used the gene signature of TREM2*^+^* TAMs to perform GSEA on RNA-seq data of 29 patients with metastatic melanoma who had been treated with anti-PD1. We identified that a TREM2^+^ TAM gene signature, including 40 genes, was overrepresented in the transcriptome of patients not responsive to immunotherapy (**Figure 4G**). These findings suggest that TREM2*^+^* TAMs are associated with immunotherapy modulation.

### 3.5 TREM2^+^ TAMs enrichment was associated with poor prognosis and potential immunotherapy modulation in ESCC

**Figure 5:**
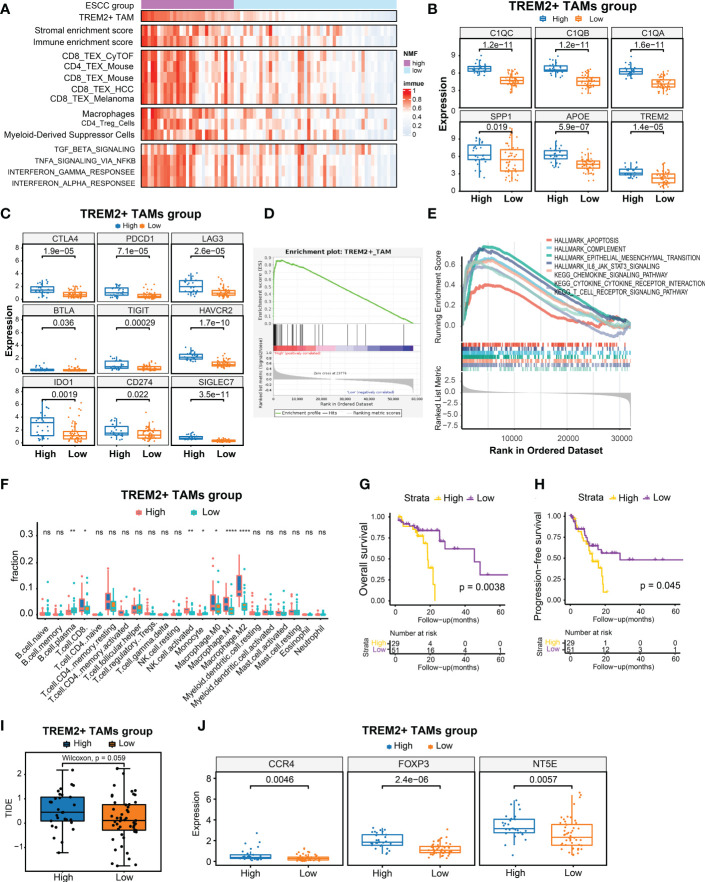
Identification of high TREM2+ TAMs-enriched group in 80 ESCC from TCGA. **(A)** Heatmap of 80 ESCC ordered by enrichment score of TREM2+ TAM gene signature, showing enrichment scores of immune, stromal, T cell exhaustion signatures, immunosuppressive cells, and T cell exhaustion related pathways. **(B)** Boxplot showing differential expression level of TREM2+ TAM signature genes between High and Low group. **(C)** Boxplot showing differential expression level of 9 immune checkpoint genes between two groups. **(D, E)** Gene set enrichment analysis for TREM2+ TAM gene signature **(D)** and hallmark gene sets and KEGG pathways between two groups. **(F)** Comparison of cell fraction inferred by CIBERSORT between two groups. **(G, H)** Kaphan-Meier estimates of overall survival and progression-free survival according to high TREM2+ TAMs-enriched group. **(I)** Boxplot showed prediction scores generated by TIDE between two groups. One-side Wilcoxon. **(J)** Expression analysis of genes related to immunotherapy resistance between high TREM2+ TAMs-enriched and low groups. All statistical significances of two classes were computed by Wilcoxon rank-sum test; ns, P >0.05; *, P < 0.05; **, P < 0.01; ****, P < 0.0001.

scRNA-seq analysis demonstrated that TREM2*^+^* TAMs were associated with immunosuppressive TME in ESCC and immunotherapy modulation. Previous studies have suggested that TREM2*^+^* TAMs could promote tumor progression in renal tumors (40) and impede the antitumor immune response in a mouse tumor model (41). To further validate its clinical relevance in ESCC, we used the gene signature representing TREM2*^+^* TAMs to calculate the enrichment score based on the bulk RNA-seq data of 80 patients with ESCC by ssGSEA (30). We also calculated the enrichment scores for another 14 signatures related to immune cells, stromal components, Tex, immunosuppressive cells, or Tex-associated pathways using the same approach. Moreover, we conducted hierarchical clustering analysis on 80 ESCC patients using the enrichment scores of 14 signatures related to immune cells, stromal components, Tex, immunosuppressive cells, or Tex-associated pathways and identified 29 patients with relatively high enrichment scores clustered together. Consequently, we defined the top 29 patients (36.25%) with high TREM2^+^ TAM enrichment scores as the high TREM2^+^ TAMs-enrichment group (High abundance group) and the remaining patients as the Low abundance group (**Figure 5A**). As expected, the High abundance group demonstrated upregulated *SPP1*, *APOE*, *TREM2*, *C1QA*, *C1QB*, and *C1QC* gene expression (**Figure 5B**). The Tex marker genes, i.e., *CTLA4*, *PDCD1*, *LAG3*, *BTLA*, *TIGIT*, *HAVCR2*, *IDO1*, *CD274*, and *SIGLEC7*, were also significantly upregulated in the High abundance group (*P* < 0.05) (**Figure 5C**). To contrast the molecular features of the TME in patients from the High and Low abundance groups, a whole-transcriptome comparison was conducted, which revealed 1121 upregulated genes with a log2FC > 1 and P < 0.05 in the High group (**Supplementary Table S9**). GSEA analysis indicated that the TREM2^+^ TAM gene signature was significantly enriched in the High group (**Figure 5D**). When examining the activated pathways in the High abundance group, GSEA indicated that 26 KEGG pathways and 11 MSigDB hallmarks were significantly enriched (FDR < 0.05) (**Supplementary Figure S4**). In this respect, epithelial-mesenchymal transition, associated with immunotherapy resistance (22, 47), was significantly enriched in the High abundance group, suggesting immunotherapy resistance in such patients. It is widely thought that severely exhausted T cells may undergo apoptosis (48, 49). Our analysis consistently demonstrated that apoptosis hallmarks were significantly enriched in the High abundance group (**Figure 5E**), suggesting that TREM2^+^ TAMs enrichment may promote severe Tex. To explore infiltrating immune cells in the High abundance group, the absolute fractions of immune cells estimated by the CIBERSORT algorithm were compared between the High and Low abundance groups. We observed that the High abundance group had significantly higher cell fractions of immunosuppressive cells, such as activated NK cells and M2 macrophages (**Figure 5F**), further indicating that TREM2^+^ TAMs enrichment is associated with an immunosuppressive TME. Kaplan-Meier estimates revealed that patients in the High abundance group had significantly poor OS (**Figure 5G**, *P* < 0.001) and PFS (**Figure 5H**, *P* < 0.05).

We also predicted the immunotherapy response using the TIDE algorithm (39) and observed that the High abundance group had a higher TIDE prediction score (**Figure 5I**). Higher TIDE prediction scores are typically associated with worse ICB responses. We compared the expression levels of *NT5E, CCR4*, and *FOXP3*, which have been linked to immunotherapy resistance, between the High and Low abundance groups (50), and revealed that the High abundance group exhibited higher expression of these genes (**Figure 5J**). In addition, we collected 10 samples from ESCC patients treated with immunotherapy for multiplex immunohistochemical staining to assess the infiltration levels of TREM2^+^ TAMs. The results showed that patients who were not sensitive to immunotherapy showed have higher density of TREM2+TAMs infiltration (**Supplementary Figure S**5).Overall, a high enrichment score of the TREM2^+^ TAMs gene signature predicted poor OS and PFS and was associated with potential immunotherapy modulation.

## 4 Discussion

It is widely acknowledged that the immunosuppressive components in the tumor microenvironment considerably impair the efficacy of immunotherapy and worsen the prognosis of malignancies (7, 51). As a crucial component of the TME (12), TAMs exhibit high plasticity and heterogeneity, enabling them to fulfill varied roles in tumorigenesis, progression, angiogenesis, and metastasis (7, 8). High TAM infiltration is strongly associated with poor tumor survival and may hijack anti-PD1 antibodies, leading to immunotherapy resistance (9, 10). Although targeting specific TAM populations is an attractive novel approach in cancer immunotherapy, the practical clinical applications are hampered by the heterogeneity of TAMs (7, 15). scRNA-seq can distinguish different cell subpopulations and explore the cellular and molecular characteristics contributing to an immunosuppressive TME (52, 53).

In recent years, researchers have emphasized the role of myeloid cells, including TAMs, in various pathological processes, with TREM2 as a major pathological-induced immune signaling hub (54). Using different scRNA-seq datasets of ESCC immune cells, we successfully identified and validated a novel TREM2^+^ TAM subpopulation that characteristically overexpressed *TREM2*, *SPP1*, *APOE*, *C1QA*, *C1QB*, and *C1QC*, with an immunosuppressive phenotype. We found that the TREM2^+^ TAMs were associated with increased CD8 Tex and Tregs, which suppressed the antitumor immune response. In a single-cell study of the breast tumor microenvironment, TREM2^+^ TAMs were found to be a kind of recruited or resident M2-type macrophages expressing genes like *SPP1, APOE*, and *C1Q*, as in our study, suggesting that the TREM2^+^ TAM subpopulation may be functionally closer to M2 polarized macrophages (55).

*C1QA*, *C1QB*, and *C1QC* are critical genes of the complement system correlated with M2 macrophage infiltration in ESCC and have been associated with poor survival (56). It has been reported that the expression of C1Q favors tumor progression, metastasis, and poor prognosis (57). When activated by communications between TAMs and cancer cells, the classical complement pathway is a crucial inflammatory mechanism that promotes the occurrence and development of cancers, increasing cancer cell proliferation and altering the immune profile of tumor-infiltrating leukocytes suppressing CD8^+^ TIL function (58, 59). Of all the complement components that may promote cancer, C1q chains, C5a and C3-derived fragments, which can inhibit antitumor T-cells response *via* the recruitment and activation of immunosuppressive cell subpopulations like Tregs and M2 TAMs (60), are possibly the most dominant lubricants of tumor progression (61, 62). Lubka T et al. (58) found that ablation of C1q, C4, or C3 contributes to the inhibition of tumor growth in mice renal cell carcinoma model. In addition, the high densities of tumor-infiltrating C1q^+^ TAMs in clear cell renal carcinoma (ccRCC) patients are significantly associated with an immunosuppressive microenvironment, which is characterized by the high expression of immune checkpoints (i.e., PD-1, LAG-3, PD-L1, and PD-L2). Co-expression of multiple immune checkpoint receptors has been associated with severe T cell exhaustion and immune resistance (63–65). Our study built upon these findings and proposed that the TREM2*^+^* TAM subpopulation could serve as an alternative origin or result of complement activation, resulting in the blockade of the cancer immunity cycle.

There is a rich literature available substantiating that SPP1 derived from macrophages promotes cancer malignancy, tumor progression, and immune escape and contributes to worse clinical course and chemo-resistance (55, 66–68). The SPP1 is involved in processes like wound healing and angiogenesis and is relevant to tumor prognosis (69). Current evidence suggests that *SPP1^+^* macrophages enhance tumor growth through TME matrix remodeling and exhibit upregulated glycolysis metabolism (70). Typically, glycolysis is the metabolic feature of M1 macrophages (85). However, it has been established that glycolysis also plays a crucial role in activating M2 tumor-associated macrophages (TAMs) (86). Moreover, SPP1 has been reported as a potential ICI inhibitor that could upregulate PDL1 on the surface of TAMs and decrease the antitumor effect of CD8^+^ T and the activation of CD4^+^T cells (67, 71). In a word, high expression of SPP1 in TAMs is related to poor prognoses and immunosuppression in cancers. *APOE* is a lipid metabolism gene associated with lipid-associated macrophage signature (72, 73). A single-cell study about clear cell renal carcinoma(ccRCC) found that *APOE* was significantly enriched in tumors from patients who recurred following surgery, identified *TREM2-APOE-C1Q*-positive macrophages infiltration as a potential prognostic biomarker for ccRCC recurrence, as well as a potential therapeutic target (40). Other studies found that *TREM2-APOE-C1Q-* positive macrophages were associated with increased markers of T cell exhaustion (74) and pro-tumor M2 macrophages such as *CD163* and MSR1 (40), which were associated with cancer clinicopathologic characteristics and outcomes (75). *APOE^-/^* ^-^ mice exhibited more resistance against tumor progression compared with wild-type mice and experienced better responses to αPD-1 (anti-PD-1) immunotherapy, and inhibition of APOE (using inhibitors such as *COG 133TFA, αAPOE*) could curb carcinoma development and foster regression when combined with αPD-1 therapy (76). These findings may help explain why some ESCC patients have poor prognoses and immune resistance, while selected ESCC patients may benefit more from adjuvant immunotherapy targeting SPP1 and/or APOE.

Subsequent analysis based on patient-derived specimens confirmed the above findings. qRT-PCR and ELISA demonstrated that the six genes were overexpressed in the plasma and tissues of patients with ESCC at our center. Furthermore, multiplex immunofluorescence successfully visualized TREM2^+^ TAMs in ESCC tissues, and a high level of infiltrating TREM2^+^ TAMs was correlated with poorer overall survival. Moreover, scRNA-seq detected a similar TREM2^+^ TAMs subpopulation associated with recurrence in renal tumor macrophages. Except for *SPP1*, the expression of *TREM2, APOE, C1QA, C1QB, and C1QC* was high in renal tumors (40). A similar TAM subpopulation expressing *C1QC*, *C1QB*, and *TREM2* was identified in colon cancer and defined as *C1QC^+^* TAMs (77). TREM2*^+^* TAMs have also been associated with Tex in a mouse tumor model, and *TREM2* deficiency or anti-*TREM2* mAb treatment curbed tumor growth in mice and improved tumor sensitivity to immunotherapy (18, 41, 42). Overall, TREM2^+^ TAMs may be present in a wide range of tumors and related to the suppression of antitumor immune response but demonstrate slightly different molecular expression characteristics.

To investigate whether TREM2^+^ TAMs impact immunotherapy response, we re-analyzed scRNA-seq data of melanomas treated with ICB therapy and observed that TREM2^+^ TAMs were significantly enriched in melanomas not responsive to ICB therapy. TREM2^+^ TAMs from ESCC exhibited a consistent expression pattern and identical signature genes (*TREM2, SPP1, APOE, C1QC, C1QB*, and *C1QA*) with melanoma cells, suggesting that TREM2^+^ TAMs were associated with immunotherapy resistance in ESCC. Moreover, GSEA indicated that the TREM2^+^ TAMs gene signature of ESCC was enriched in melanomas not responsive to anti*-PD1* immunotherapy. In summary, a high abundance of TREM2^+^ TAMs was associated with potential resistance to immunotherapy, making it a potential target for enhancing the response to antitumor immunotherapy.

To further validate the clinical relevance of TREM2^+^ TAMs, TCGA ESCC data were used to explore associations with TREM2*^+^* TAMs gene signature. Based on 80 ESCC RNA-seq samples, we calculated the enrichment scores of TREM2*^+^* TAMs gene signature and other Tex-related signatures and identified 29 patients with high TREM2*^+^* TAMs enrichment scores. As expected, these patients had higher expression levels of *TREM2*, *SPP1*, *APOE*, *C1QA*, *C1QB*, and *C1QC*. Similarly, nine inhibitor receptors representing Tex were upregulated in the High abundance group, suggesting that high TREM2*^+^* TAMs enrichment was associated with Tex in the ESCC cohort, consistent with our scRNA-seq analysis results. GSEA showed that apoptosis, complement, and epithelial-mesenchymal transition hallmarks were activated in patients in the High group, which have been associated with immunosuppression (78, 79). In addition, the chemokine signaling pathway and T cell receptor signaling pathways were activated. GSEA revealed that TREM2^+^ TAMs might regulate these pathways, impacting immunosuppression. Furthermore, survival analysis showed that patients in the High abundance group had poorer OS and PFS, consistent with a previous report that TREM2^+^ TAMs are associated with recurrence in renal tumors (40). These results suggest that the high infiltration of TREM2^+^ TAMs contribute to Tex and an immunosuppressive TME, leading to poor clinical outcome. Moreover, based on the expression data of the 80 TCGA patients with ESCC, TIDE predicted that the High abundance group was more resistant to immunotherapy. Furthermore, patients in the High abundance group exhibited overexpression of *IDO1, NT5E, CCR4, and FOXP3*, which have been associated with immunotherapy resistance. Patients with upregulated immune checkpoints usually experience better survival after ICB treatment. However, some studies have reported contradictory findings, indicating that co-expression of multiple immune checkpoints could result in T cell depletion and resistance to immune therapy (63–65). Overall, high enrichment scores of the TREM2^+^ TAMs gene signature can predict poor outcomes and potential immunotherapy modulation.

We also determined that TREM2*^+^* TAMs highly expressed soluble factors such as *SPP1*, *APOE*, *C1QA*, *C1QB*, and *C1QC*, which suggested that this TREM2^+^ TAMs subset may be exocrine or paracrine for establishing the microflora critical for cancer cell invasion and the metastasis environment. Despite the TCGA database showing that high *TREM2*, *SPP1*, *APOE*, *C1QC*, *C1QB*, and *C1QA* expression leads to poor OS, the roles of these genes in ESCC remain unclear. Complementing traditional biopsies with noninvasive liquid biopsies is one of the most exciting breakthroughs in cancer diagnostics (80). The genes characteristically expressed in the TREM2^+^ TAM subpopulation encoded secretory proteins and were validated *via* ELISA in plasma samples from our center (**Figure 3B**). Consistently, previous studies have shown that SPP1, APOE, C1QA, C1QB, and C1QC are significantly elevated in the plasma of patients with cancer (81–83). Overall, the above genes exhibit huge potential as targets for noninvasive liquid biopsies for ESCC detection.

Our study also has some limitations. Although our work is primarily computational and omics-based, we used multiplex immunofluorescence to detect TREM2^+^ TAMs and validate the specific signature genes by qRT-PCR and ELISA. Nevertheless, further clinical validation is required to establish the correlation between TREM2^+^ TAMs and immunotherapy modulation in ESCC.

To summarize, our study substantiated the presence of TREM2^+^ TAMs and their molecular signature as predictors of ESCC prognosis and response to antitumor immunotherapy. Our findings suggest that signature genes associated with TREM2^+^ TAMs, including APOE, SPP1, C1QC, C1QB, and C1QA, can potentially serve as biomarkers for liquid biopsies and immunotherapy modulation in ESCC.

## Data availability statement

The original contributions presented in the study are included in the article/**Supplementary Material**. Further inquiries can be directed to the corresponding authors.

## Ethics statement

This study was approved by the Sun Yat-sen University Cancer Center ethics committee (GZR2018-120). The patients/participants provided their written informed consent to participate in this study.

## Author contributions

HL, MY, YM and LZ: Conceptualization, data curation, formal analysis, writing–original draft, writing–review and editing. SF, YX, LT, CW, XZ, LZ, LG, HD: data-collecting, writing–review and editing. MY, ZW and HW: Conceptualization, supervision, funding acquisition, writing–original draft, project administration, writing–review and editing. All authors contributed to the article and approved the submitted version.
