# Supplementary material for: Identification of TREM2-positive tumor-associated macrophages in esophageal squamous cell carcinoma: implication for poor prognosis and immunotherapy modulation
Source: Front Immunol. 2023 Apr 28;14:1162032. doi: 10.3389/fimmu.2023.1162032 (PMC10175681; doi:10.3389/fimmu.2023.1162032)

**Supplementary Figure S1**. Identification and characterization of TREM2+ TAM subpopulation in ESCC. **A**, UMAP plot showing expression level of C1QA, C1QB, C1QC, APOE and SPP1 in each cell type. **B**, Pathway and process enrichment analysis for differentially expressed genes of C1Q+ macrophage between tumor and adjacent normal tissue. **C**, Violin plot showing expression level of T cell exhaustion markers and Treg markers. **D**, Dotplot showing significant ligand-receptor pairs mediating cell-cell interaction; color represents communication probability, size represents static significance.

**

**


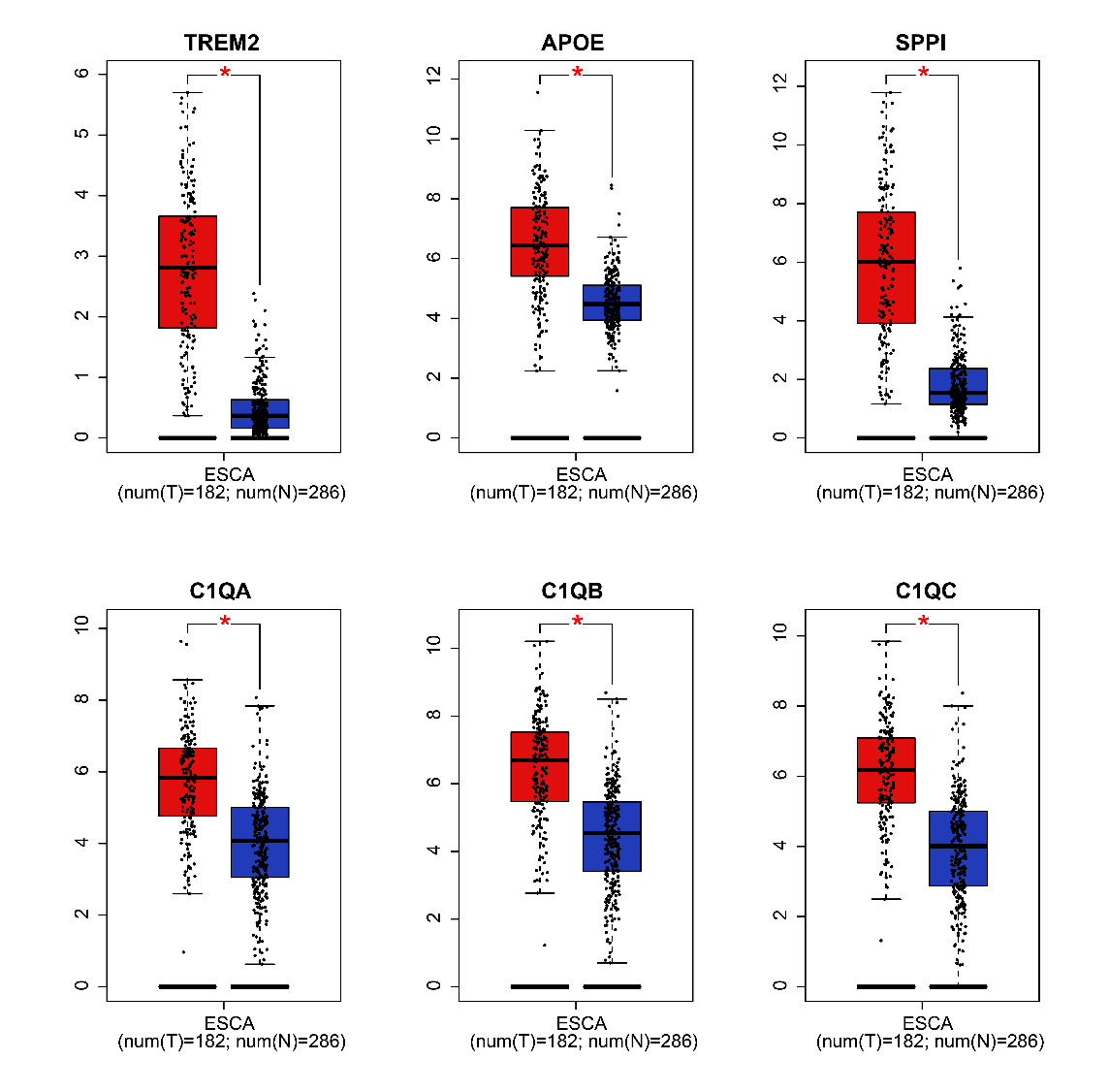
**Supplementary Figure S2.** TCGA and GTEx data confirmed that SPP1, APOE, C1QA, C1QB, and C1QC were highly expressed in esophageal cancer.

**Supplementary Figure S3.** Kaplan-Meier Plotter online database analysis showed that SPP1, APOE, C1QA, C1QB, and C1QC were associated with the poor prognosis of esophageal squamous cell carcinoma.

**
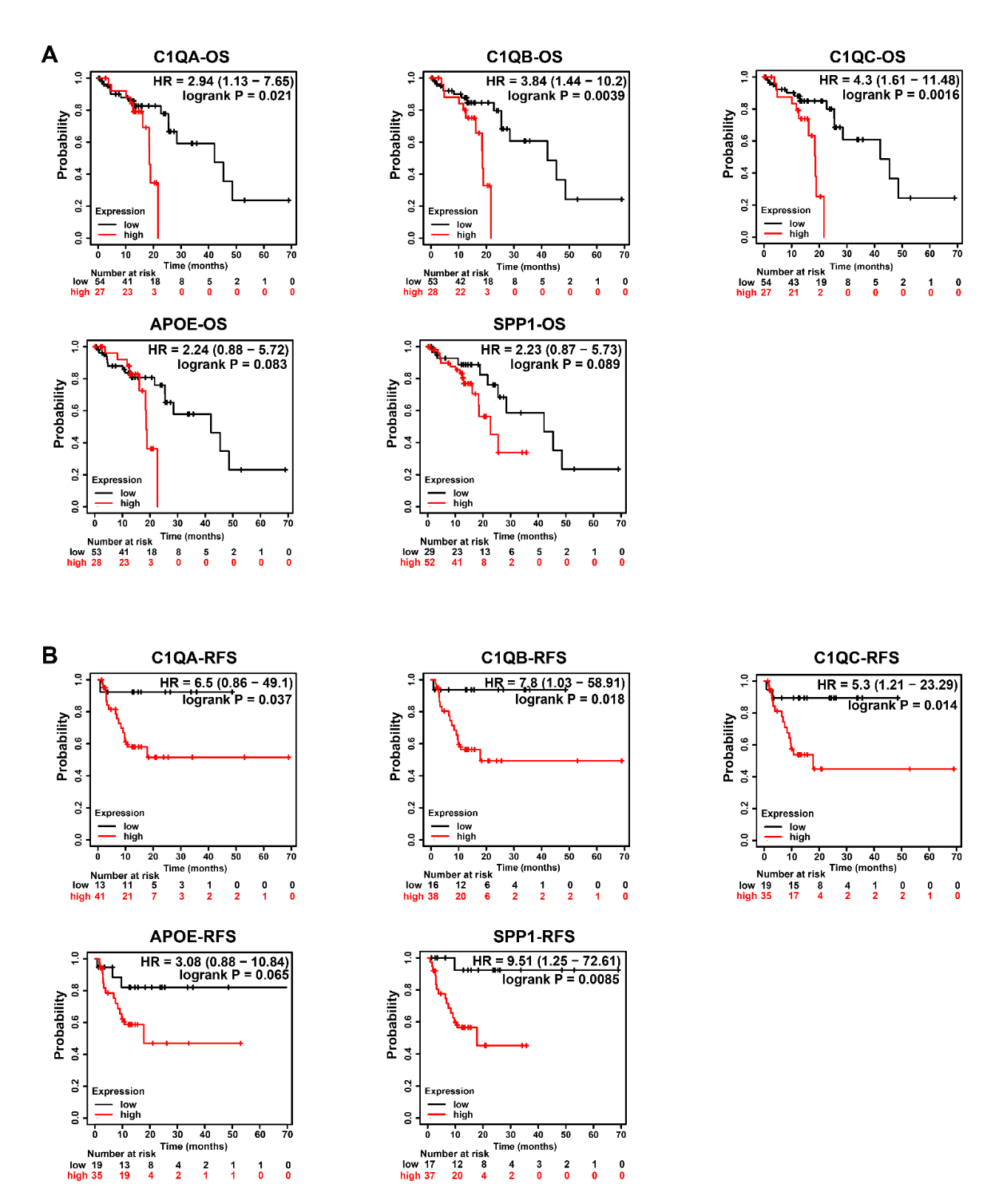
**

**Supplementary Figure S4.** Gene set enrichment analysis on KEGG pathways and Hallmark gene sets.


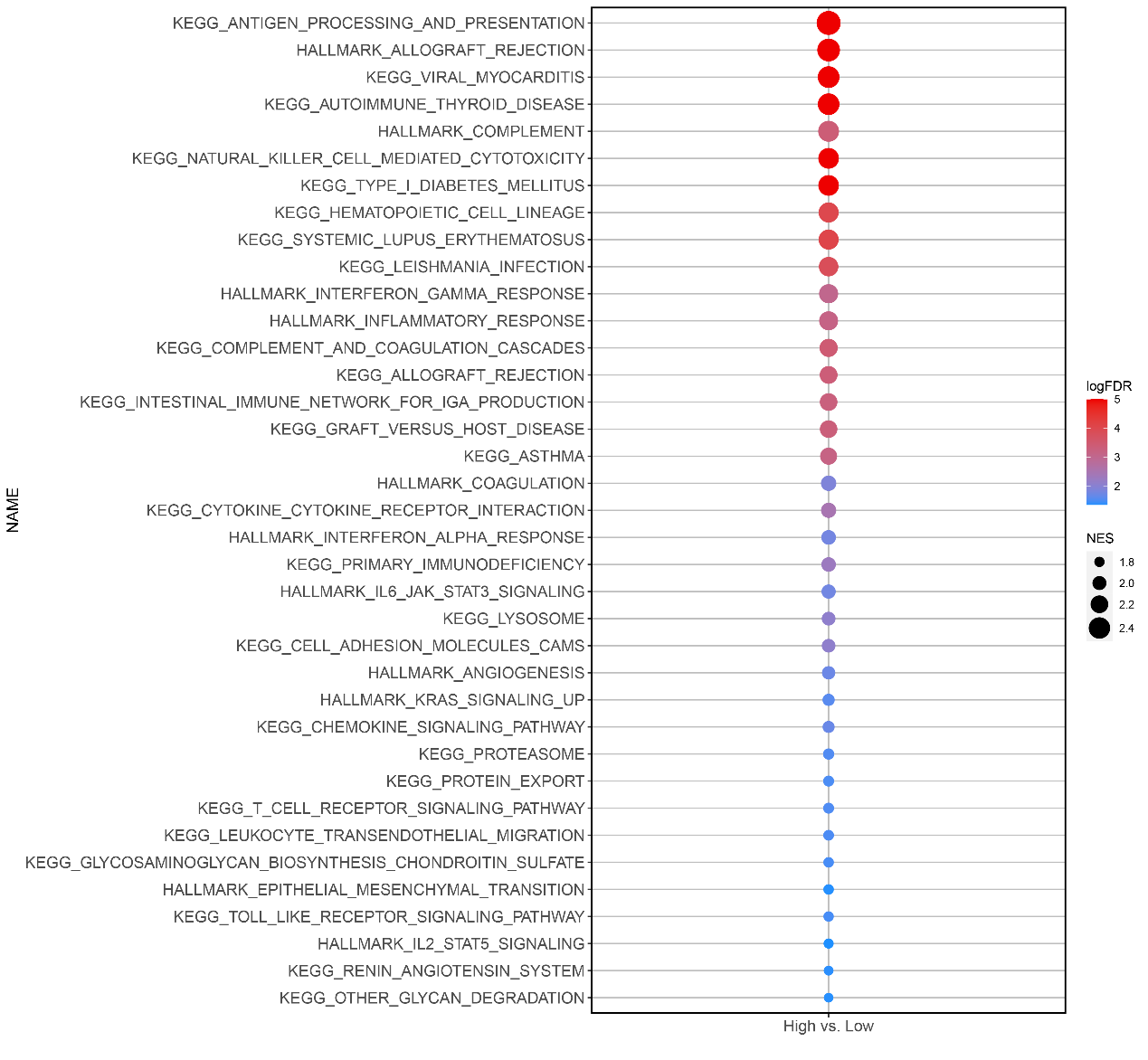


**Supplementary Figure S5.** TREM2^+^ TAMs cell abundance in ESCC patients between sensitive and not sensitive to immunotherapy.


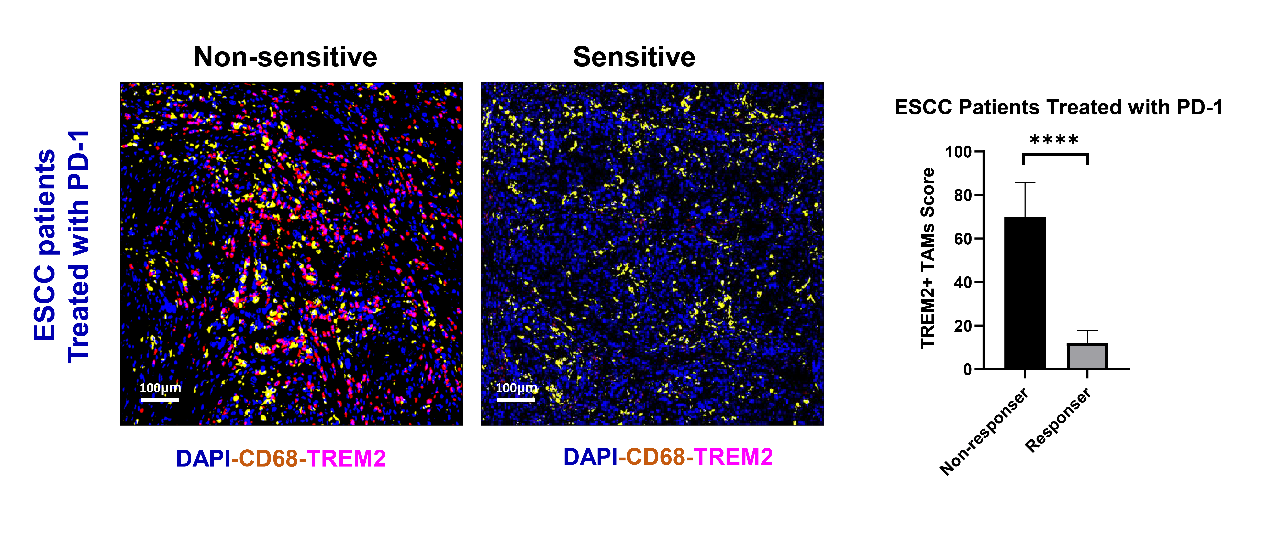

Supplement: Supplementary file 1 [file DataSheet_1.docx]
